# Research on Taxiway Path Optimization Based on Conflict Detection

**DOI:** 10.1371/journal.pone.0134522

**Published:** 2015-07-30

**Authors:** Hang Zhou, Xinxin Jiang

**Affiliations:** Nanjing University of Aeronautics and Astronautics, College of Civil Aviation, Nanjing, 210016, Jiangsu, China; Southwest University, CHINA

## Abstract

Taxiway path planning is one of the effective measures to make full use of the airport resources, and the optimized paths can ensure the safety of the aircraft during the sliding process. In this paper, the taxiway path planning based on conflict detection is considered. Specific steps are shown as follows: firstly, make an improvement on A * algorithm, the conflict detection strategy is added to search for the shortest and safe path in the static taxiway network. Then, according to the sliding speed of aircraft, a time table for each node is determined and the safety interval is treated as the constraint to judge whether there is a conflict or not. The intelligent initial path planning model is established based on the results. Finally, make an example in an airport simulation environment, detect and relieve the conflict to ensure the safety. The results indicate that the model established in this paper is effective and feasible. Meanwhile, make comparison with the improved A*algorithm and other intelligent algorithms, conclude that the improved A*algorithm has great advantages. It could not only optimize taxiway path, but also ensure the safety of the sliding process and improve the operational efficiency.

## Introduction

With rapid development of Chinese civil aviation industry, the number of runways is increasing, taxiway system tends to be increasingly perfect, and aircraft ground operation paths are becoming more and more complex. In order to ensure safety and improve operational efficiency, the first is to make taxiway path pre-planning, which plays an important role in the airport resource distribution. Thus, achieve airport resource use effectively and alleviate flight delays. A lot of prior literatures on taxiing path planning has been devoted to solving the problem. Teixeir (1992) considered the arrival and departure aircraft respectively on the condition of single runway, by setting priorities for the arrival aircraft to optimize runway scheduling algorithm combined with functions [1]. Hesselink (1998) used improved Dijkstra path planning algorithm to provide a reference to the approach path for aircraft combined with the geographic information provided by the airport road network map. However, it was simulated in the static environment, without considering the possibility of dynamic conflict during the process of operation [2]. Tenenuaum (2000) proposed genetic algorithm to solve the problem of taxiway optimization scheduling [3]. Smeltink (2004) applied mixed integer linear programming model to deal with the aircraft movement problems in the flying area of the Airport Schiphol. He regarded the conflict delay as the optimization target, and finally determined the taxiing path after optimization [4]. Marin (2006) put forward the concept of linear multi-object flow network to describe the constrains of the taxiway flow based on the contradiction of the number of aircraft and airport capacity [5]. G. Clare (2009) applied relaxation algorithm to establish MILP equation without consideration the conflict of aircraft in the initial calculation, then added one constrain after every calculation, and got the optimal solution after several iterations [6]. The domestic research on airport taxiway path planning started late, but in recent years, with the development of civil aviation, many scholars had done a lot of excellent research achievements on the taxiway model building, which had been used to searching for the shortest optimization paths. XZ (2011) Combined the algorithm of a signal object and A* algorithm to improve the speed. By comparing the experimental results, verification the search efficiency of improved algorithm than the standard A* increased 14.7% [7]. YY (2014) analyzed the problem of obstacle avoidance shortest path, compared the advantages and disadvantages with A* and Dijkstra algorithm. In the simulation environment of electronic maps, the A* algorithm was applied to realize obstacle avoidance shortest path from the starting position to the target position [8]. NL and QZ (*et*.*al* 2012) established a taxiway path optimization model by considering safety separation, sliding rule and conflict-free as constraints. The results indicated that the taxiway scheduling model and the optimal algorithm were feasible [9]. ZL and HG (*et*.*al* 2008) proposed two novel scheduling algorithms for solving the problems based on genetic algorithm. A graph model and its corresponding matrix coding were presented to pave the way for the taxiway scheduling problem. Experimental results validated the feasibility of the proposed model and algorithms [10]. XZ and XT (*et*.*al* 2013) divided the airport surface into typical operation units, such as taxiway intersection and line segment. A modular surface operation model was built based on the extended timed place Petri net (ETPPN), and the genetic algorithm was applied to solve the model. The results demonstrated that the traffic situation could be described more closely to the real surface operation [11]. XT and YW (*et*.*al* 2010) built the general framework including static planning, dynamic planning and on-line updating taxiway paths to implement dynamic taxiway paths planning for aircraft in an advanced surface movement guidance and control system (A-SMGCS) [12]. TD and JP (*et*.*al* 2010) presented an airport taxiing scheduling optimization strategy based on genetic algorithm [13]. JZ and XX (*et*.*al* 2014) designed a multi-Agent model to meet the aircraft rules and controllers’ experience and the Agent reasoning process was described by using event-condition-action language. Finally, the model was certified with the Anylogic simulation platform, and the results showed the regular algorithm can not only avoid aircraft conflicts effectively and intelligently, but also suit the aircraft operation process [14]. YD and RA (2011) studied the optimization model with the constraint of the safety interval, taxiing rules and conflict-free and verified the feasibility and advantages of the model [15]. LX (2012) discussed several possible aircraft taxiing conflict situations and introduced two corresponding resolution strategies. And the airport surface conflict resolution strategy based on holding at taxiway was studied [16]. CW (2012) used 3DMax to build a scene model based on Petri net, with sliding time constraint to achieve real-time simulation technology of airport aircraft operation by plane flow simulation [17]. GG (2012) established a model by considering the safety separation, taxiing rules and conflict-free as constraints, the ant colony algorithm was given for arrival and departure flights which provided decision support for safe operation in busy airport [18].

Although many scholars have done the research of the airport surface operation, some researches without integrating conflict avoidance strategy after the path being established, or without considering the direction of the aircraft operation. On the basis of existed researches, in this paper, an improved A* algorithm based on A* algorithm for solving the taxiway path planning problem combined with conflict detection is proposed. As is well known, A* algorithm is usually used for searching shortest path, then conflict avoidance strategy is added into this process to establish a path collection. Conduct simulation experiment to verify the feasibility of the model. We also apply genetic algorithm and ant colony algorithm, which are well-known intelligent algorithms, to compare with improved A* algorithm results.

## Model and Methods

### Taxiway system modeling and conflict analysis

#### Taxiing system network architecture

Taxiway plays an important role in the airport surface operation system, which connects the runway system and gate position system. In this paper, make initial optimization path planning for the taxiway system, mainly contains making plan and optimization of aircraft sliding strategy on the airport movement area. The runway system, taxiway system and gate position system are abstracted to a geometric network diagram which is composed of links and nodes. The runways and taxiways are regarded arcs; their intersection points and starting point (including the gate position and runway ending) are regarded as nodes.

**Fig 1:**
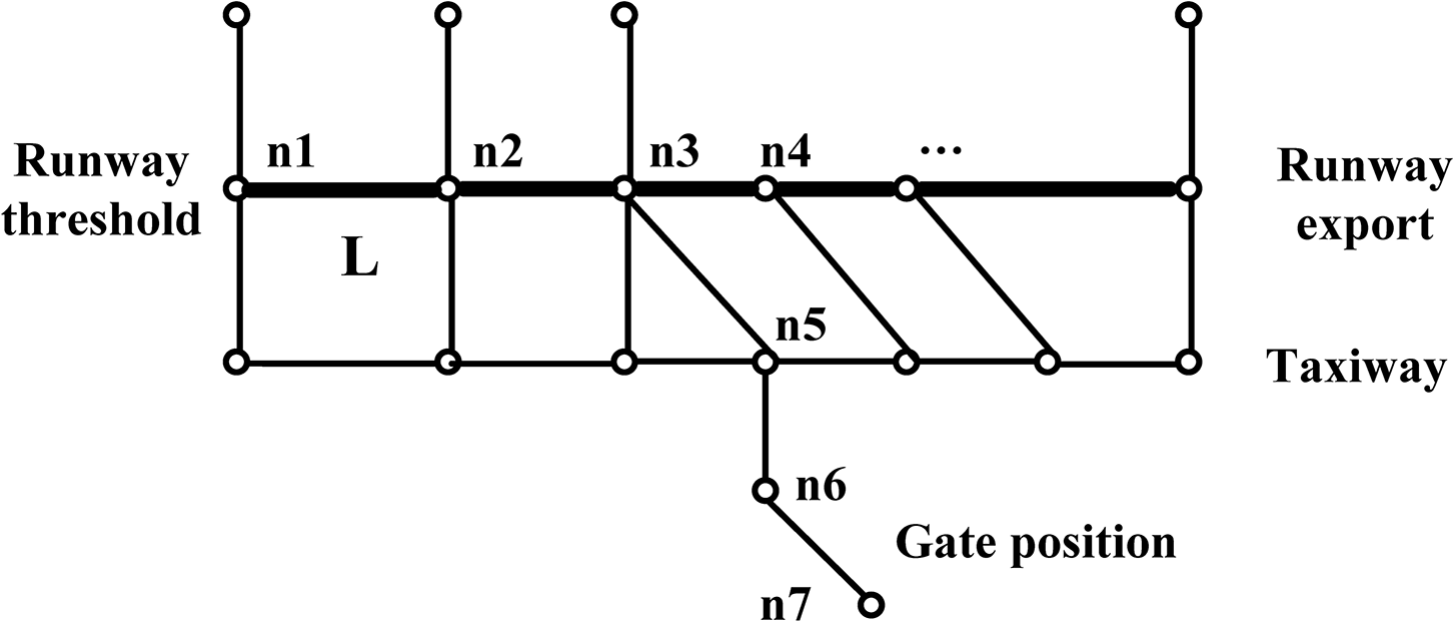
Taxiing system diagram.

The basic airport taxiway system layout is given in Fig 1. Node represents the intersection of taxiways and runways, taxiways and taxiways, a line segment with direction represents sliding path among all nodes. So the whole taxiing system can be simplified into a directed network diagram. *N* is the collection of all nodes, *L* is the collection of all links. In the process of sliding, aircraft movement has its direction, means that it can slide at this path in both directions. In order to express this situation through the network map, it can be indicated by two nodes with a pointing arrow. So in the Fig 1, the link *L* from left to right can be expressed as *n*1→*n*2, while from right to left is *n*2→*n*1.

For each aircraft, sliding path can be represented by a series of arrows and node number. For example, *n*1→*n*2→*n*3→*n*5→*n*6→*n*7 shows the process that the aircraft sliding on the runway and then getting into the taxiway at the node *n*3, and finally arriving at the gate position. This is a complete sliding path, and each aircraft has many possible paths. What discussed in this paper is to find out the sliding path without conflict and with the shortest distance as well.

#### Taxiway conflict analysis

Taxiway is a necessary bridge connecting runways and gate position system, and plays an important role in the whole airport operation. There are three kinds of conflict may occur during the process of sliding:

1. **Intersection point conflict.** There are equal or greater than two aircraft passing through one after another at the same intersection point, and time interval does not meet minimum safety interval.
  Suppose that there are two aircraft *A* and *B* sliding on the taxiway, their safety interval standard is *L*, running speed is *V*_*a*_ and *V*_*b*_, they all need to pass the node *N*, the time arriving at the node *N* is *T*_*a*_ and *T*_*b*_ respectively. If
  $$\left|T_{a}-T_{b}\right|\leq L/\left|V_{a}-V_{b}\right|$$ (1)
  then these two aircraft do not meet the minimum safety standard at the intersection point, so there is the intersection conflict.
2. **Head conflict.** On the same taxiway, there are two aircraft, but their predetermined taxiing path is overlapping and run in the opposite direction, there may be a conflict.
  Suppose that there are two aircraft *A* and *B* sliding on the taxiway, they run in the opposite direction and do not change their running direction before encounter, so there will be a head conflict. It is the most dangerous one in the taxiway system that could lead to crash, causing incalculable consequences.
3. **Tailgating conflict.** On the same taxiway, there are two aircraft, but their predetermined sliding path is overlapping and run in the same direction. Meanwhile, the later aircraft runs faster than the former one; there may be a tailgating conflict.
  Suppose that there are two aircraft *A* and *B* sliding on the taxiway, *A* is behind *B*, their safety interval standard is *L*, running speed is *V*_*a*_ and *V*_*b*_, and run in the same direction. Besides, they all need to pass the node *N*, the time arriving at the node *N* is *T*_*a*_ and *T*_*b*_. Because the aircraft *A* passing the node *N* after the aircraft *B*, the time difference is (*T*_*a*_−*T*_*b*_), during this period, the sliding length of aircraft *B* is (*T*_*a*_−*T*_*b*_)×*V*_*b*_, it is also the distance difference *s*' between *A* and *B*. If *s*' ≤ *L*, the subsequent sliding processing after passing the node *N*, the aircraft *A* is sure to catch up with aircraft *B*, resulting in the tailgating conflict. So, if
  $$\left(T_{b}-T_{a}\right)*V_{b}\leq L$$ (2)
  these two aircraft do not meet the minimum safety standard after the intersection point, it is possible to be a tailgating conflict. Safety interval is listed in Table 1. Different types of flights have different time interval. (Here, H represents the heavy flight, M represents the medium flight, L represents the light flight).

**Table 1:** Safety interval standard of sliding aircraft/s.

tail	lead		
	H	M	L
H	42.9	33.3	20
M	42.9	33.3	20
L	42.9	33.3	20

### A* algorithm

#### Reliability and availability analysis of A* algorithm

A * algorithm is a heuristic search algorithm to solve dynamic programming problem, which is often used for path planning. The basic idea is to set an evaluation function *f*(*n*), which represents the real value of node *n*. It is also the total liner distance of shortest practical distance from starting point to the node *n* and the distance from the node *n* to the terminal node. The smaller *f*(*n*), the higher the selectivity. The node with minimum *f*(*n*) is determined as the next extended node. If the node to be extended is the terminal node, which indicates that the path searching process has been finished, and the sliding path has been generated [19].

The A* algorithm has two properties: admissibility and homogeneity.

1. If *h*(*n*) represents the practical shortest distance from node *n* to the terminal node, and for any node *n*, there is *h*'(*n*) ≤ *h*(*n*), so the *h*'(*n*) is admissible;
2. If the node *n*_*j*_ is the successor node of the node *n*_*i*_, for all the node pairs (*n*_*i*_,*n*_*j*_) of the network diagram meet the condition *h*'(*n*_*i*_) – *h*'(*n*_*j*_) ≤ *h*(*n*_*i*_) − *h*(*n*_*j*_) (the later formula is the shortest path from the node *n*_*i*_ to the node *n*_*j*_), so it indicates that *h*'(*n*) has the homogeneity. If *h*'(*n*) meets homogeneity, then the optimal path has been find out from the starting node to the node *n* when the path expended to it.

Obviously, as for the airport taxiway system, there is no isolated node path, and the path between two nodes is limited. So there must be a shortest path satisfying both completeness and optimality that can be calculated by A * algorithm. On the other hand, each link really exists in the taxiway system, and the length is positive, so it must be able to find out the shortest path by A * algorithm.

#### Set evaluation function

Now, set the function *f*(*n*) to evaluate the real value of the node *n*. Then judge it whether it is the optimal node as the next extended node. Because *n* is one of the nodes in the entire path, so the practical distance from the node *n* to the starting node and the distance from the node *n* to the terminal node can be used to evaluate the value of the node *n*. Assume the former distance is *g*(*n*) and the later distance is *h*(*n*). If the node *n* is included in the optimal path, the practical length of the optimal path can be expressed as:

f(n)=g(n)+h(n) (3)

As for the path with the same starting node and terminal node, the smaller *f*(*n*) is, the shorter taxiing path. But which node as the node *n* is uncertain. So, it is needed to select a point as the starting node, and make evaluation of all nodes connected to it in order to pick out its successor nodes. Until the next successor node is the terminal node, the entire path is completed.

During this process, each node before the node *n* is certain, so *g*(*n*) is known. But the successor nodes are uncertain, so it is needed to set *h*'(*n*) to estimate *h*(*n*). The straight line is the shortest between two nodes, so the practical distance from the node *n* to the terminal node must be less than its straight line distance. Therefore, setting *h*'(*n*) is the straight line distance of two nodes; it is the value which is closest to the optimal path. Estimated value of the node can be calculated by:

f'(n)=g(n)+h'(n) (4)

*f*'(*n*): An estimated value of the optimal path length which the node *n* is included;

*g*(*n*): The practical distance from the starting node to the node *n*;

*h*'(*n*): The estimated value from the node *n* to the terminal node.

The basic step of the A*Algorithm is: from the starting node *A*, calculate their corresponding estimated values for all the nodes connected to the node *A* according to the evaluation function and make comparison of them; Then choose the minimum one as the successor node. Next, the successor nodes is the starting point, calculate next successor node similarly (during the calculation of the neighboring nodes, the parent nodes are not counted). Make continuously projections by this method until the terminal node *Y* is found. Finally, on the basis of the father nodes of each node, moving forward, we can find a path, which is the shortest path we are looking for.

**Fig 2:**
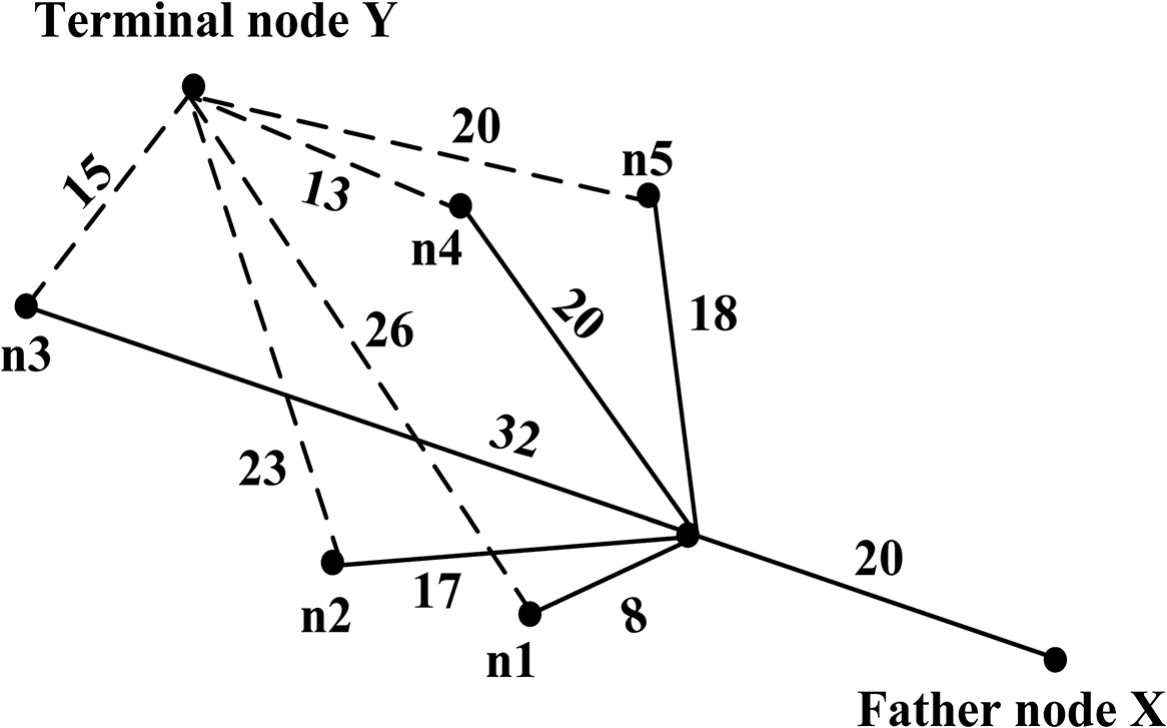
The basic idea of the A* algorithm.

Simplify the process to represent its basic idea, shown in the Fig 2.

As shown in the Fig 2, the solid line represents the real link, the dotted line represents the straight line distance from the node to the terminal node, and the number in the line represents the length of line segment. The *f*'(*n*) consists of two parts, one is *g*(*n*), which represents the practical distance from the starting node to the node *n*; the other one is *h*(*n*), which represents the straight line distance from the node *n* to the terminal node, and it is irrelevant to links. If the father node *X* is the starting node in the Fig, adjacent nodes of it are *n*1,*n*2,…*n*5. Suppose that the path from the starting node to the terminal node *Y* passing the node *n*, and the shortest path length is 25 from the starting node to the node *n*, so the function relationship between them can be represented in Table 2.

**Table 2:** Function relationship of *f*(*n*) and *h*(*n*).

The shortest distance from the starting node to the node *n*	Adjacent node	The distance from the node *n* to its adjacent node	*f*(*n*)	*h*(*n*)	Evaluation value
20	n1	8	28	26	54
	n2	17	37	23	60
	n3	32	52	15	67
	***n4***	***20***	***40***	***13***	***53***
	n5	18	38	20	58

As can be seen from the Table 2, the node with minimum evaluation value is *n*4, and its value is 53. So the node *n*4 is the successor node of the node *n*. If the node *n*4 is the terminal node, the process of searching for successor nodes has been finished, and the shortest path has been find out, that is father node *X*→node *n*→node *n*4. It is a simplified model for the basic steps of the A * algorithm.

### Improved A * algorithm

In reality, the node can be selected by multiple paths repeatedly, which may lead to aircraft conflict. Although A* algorithm has taken into account the optimal path problems, it ignores the existing risk of conflict during the sliding process. Therefore, on the basis of the A* algorithm, add conflict detection procedure into it and apply multi-objective optimization function to take measures to solve the conflict. Hence, the improved A* algorithm is created to avoid conflict, reduce fuel consumption and ensure safety.

#### Conflict detection process

First of all, the system calculates initial path according to the original information for the flight. During the process of flight sliding in the routes, the flight coming into the taxiway from different nodes, its sliding speed must be different, and the sliding process is not a uniform motion, so the sliding speed is a variable. In addition, considering the economy of flight operations and reducing the complexity of calculation, define the average sliding speed of three kinds of flight according to the type of flight, they are: heavy flight is 7m/s, medium flight is 6 m/s and light flight is 5 m/s. For the sliding aircraft, the arrival time of each node is determined by the starting time, sliding speed and the length of links. And the schedule of a node is the time reaching the parent node plus the former sliding time.

After the initial path, sliding speed, expected arriving/leaving time, and the length of the link *l* from the node *n*−1 to the node *n* have been determined, then the time *t*_*n*_ for each node passing the initial path of every flight could be calculated, add it into the node schedule, and delete it after the flight passing the node. That is

$$t_{n}=t_{n-1}+\frac{l}{v}$$ (5)

This schedule is treated as the foundation of the conflict detection process at this node. It can also be used for recording flight data. Deposited items into the schedule in order and make retrieve of the entire flight path after the first flight data has been entered, and then conducted conflict avoidance strategy to determine the final path. The items are listed in the Table 3.

**Table 3:** The node schedule.

The number of node	Flight number	Type	Arriving time *t*_*i*_	Father node	Successor node

For a certain flight, the number of nodes in initial path is *n*, written as *P*_*n*_, and the starting point is *P*_0_. Make retrieve of the schedule of the node *P*_*n*_ from *n* = 1. Set the time in the schedule is *t*_*i*_, the time flight passing this node is *t*_*n*_, *c* is the safety interval in Table 1, so

$$\left\{ \begin{array}{l} \left|t_{i}-t_{n}\right|\leq c\;there\;is\;a\;conflict\\ otherwise\;there\;is\;no\;conflict,then\;t=t+1,searching\;for\;next\;node \end{array}\right.$$ (6)

**Fig 3:**
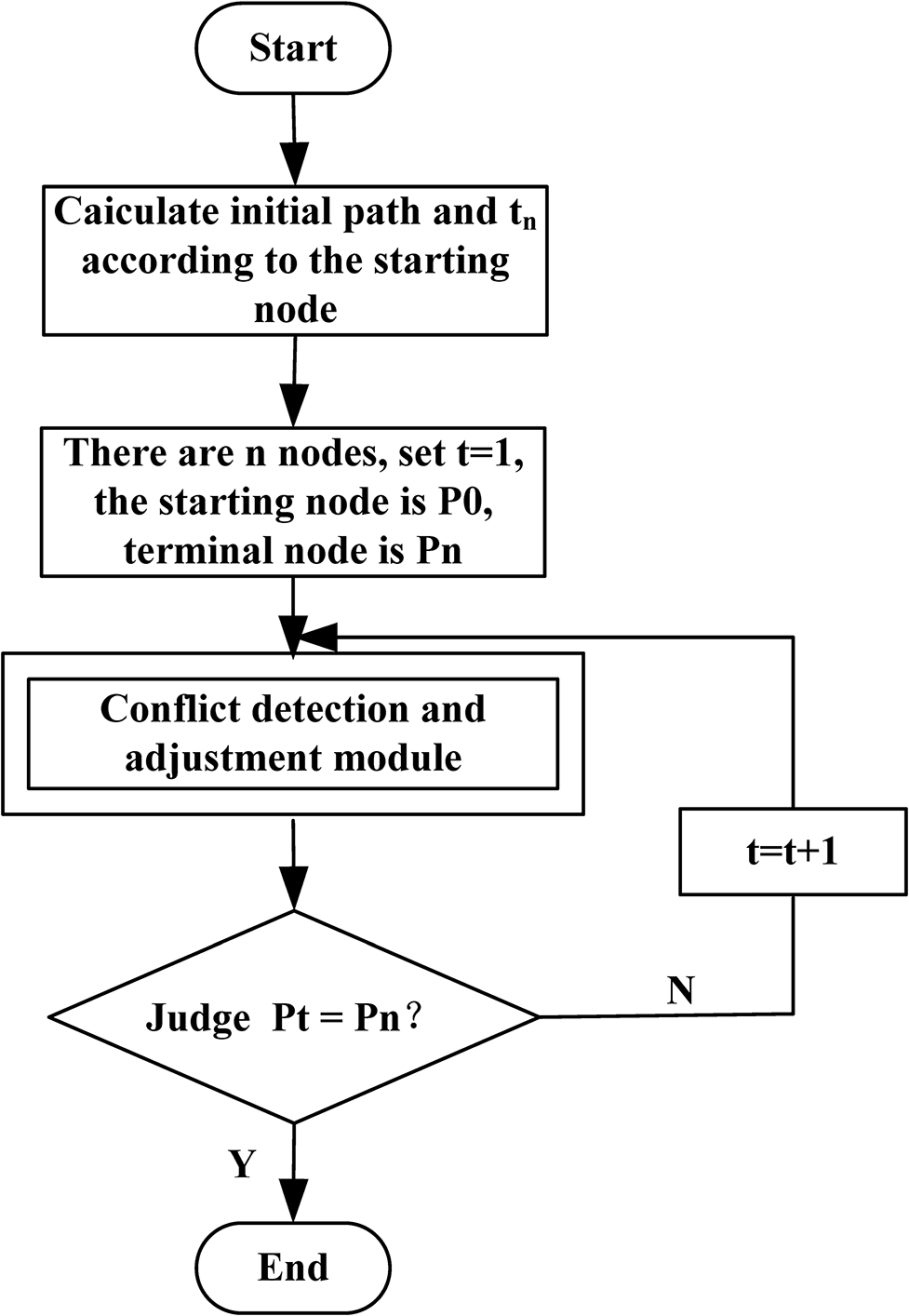
The flow chart of conflict avoidance strategy.

Fig 3 is the flow chart of conflict detection. If there is a conflict, then judge the priority of these flights. The priority principle is: large aircraft in preference to small aircraft and departure flight in preference to arrival flight. If the priority of the flight is high, it can go firstly; If the priority of the flight is low, it should apply multi-objective optimization function to determine whether it holds on the position, the waiting time is the safety interval, or looking for new sliding path.

#### Conflict avoidance-multi-objective optimization function D(m)

When there is a conflict at the node, the aircraft faced with two choices: one chooses to wait, goes through the node after the current aircraft passing; the other one, re-selects the path.

Set an evaluation function to express the expansion price of the node, written as *D*(*m*).

D(m)=g(n)*x/v+t(n)*y (7)

*D*(*m*): The expansion price of the *m*th optimal node;

*g*(*n*): The practical distance from the *m*th optimal node to the node *n*;

*t*(*n*): The waiting time for the aircraft at the *m*th optimal node;

*v*: The speed of the aircraft;

*x*: The weight coefficient of sliding consumption;

*y*: The weight coefficient of waiting consumption.

*x* and *y* are the weight coefficients of consumption cost of the aircraft sliding or waiting. In general, the consumption cost in the waiting process is much less than sliding. Every airport could set the weight coefficient flexibly according to its actual situation, so the calculated results could be closer to the real situation.

**Fig 4:**
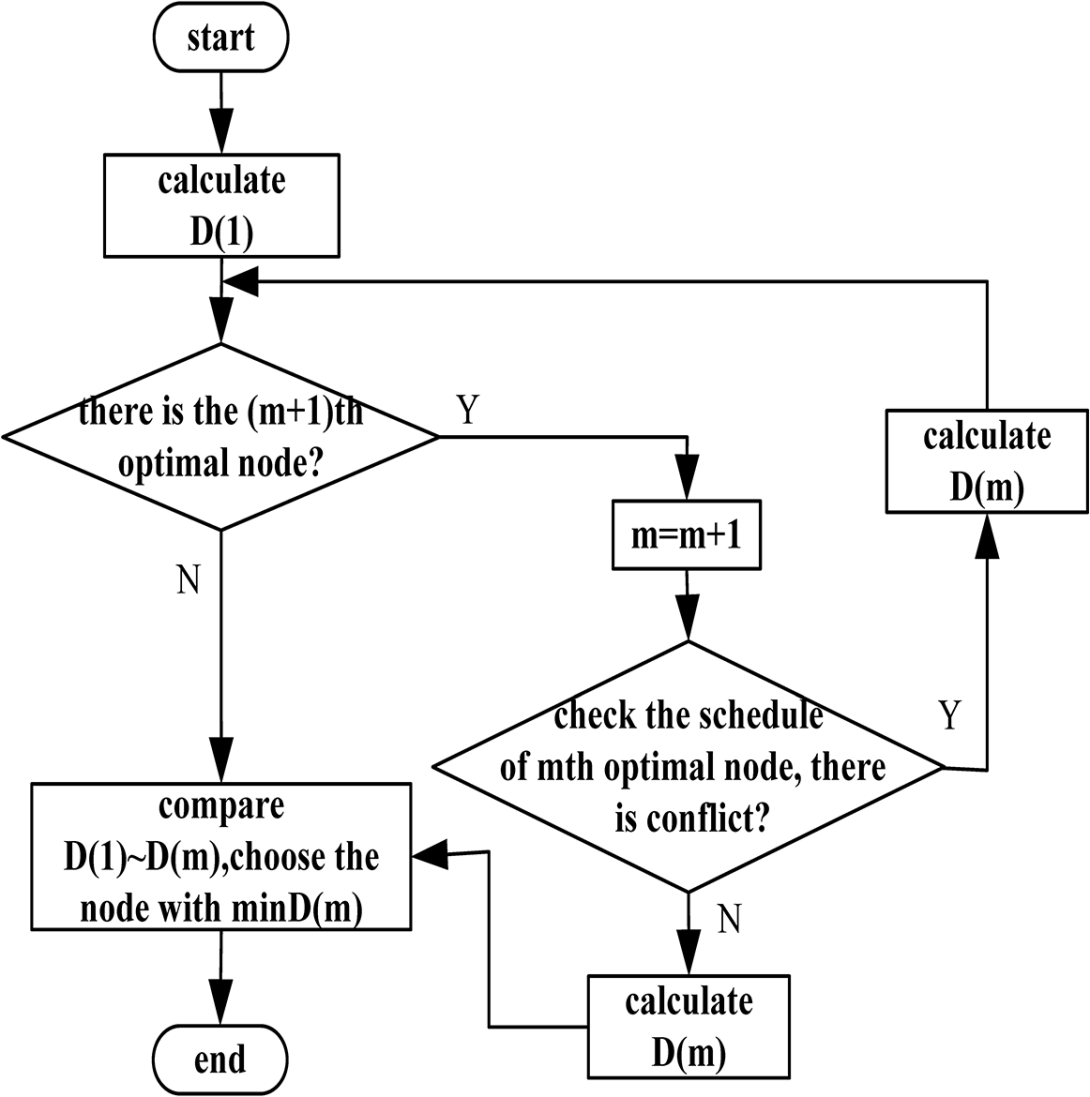
The flow chart of improved A* algorithm.

Prior to taking measures, it is needed to calculate the price of the passing node. The price of the *m*th optimal node is *D*(*m*), so the price of the most optimal node is *D*(1). Then, check whether there is a suboptimal node. If there is, *m* = *m* + 1, do the retrieval of the schedule for the *m*th optimal node. Then judge whether there is conflict or not, if there is, calculate the waiting cost at this node then turn to its suboptimal node. If there is not, calculate the price of the node, and store in the *D*(*m*) function. Repeat the process until there is no conflict in the *m*th optimal node or no suboptimal node, then evaluate the price of each node. At the same time, record *D*(*m*), and make a selection to choose the measure with min*D*(*m*). The process is shown in Fig 4.

Do the retrieval of all nodes in the path, until the terminal node is found, then the generated path is the final path without conflict. So, the conflict detection process is finished.

## Simulation and Analysis

### Simulation

**Fig 5:**
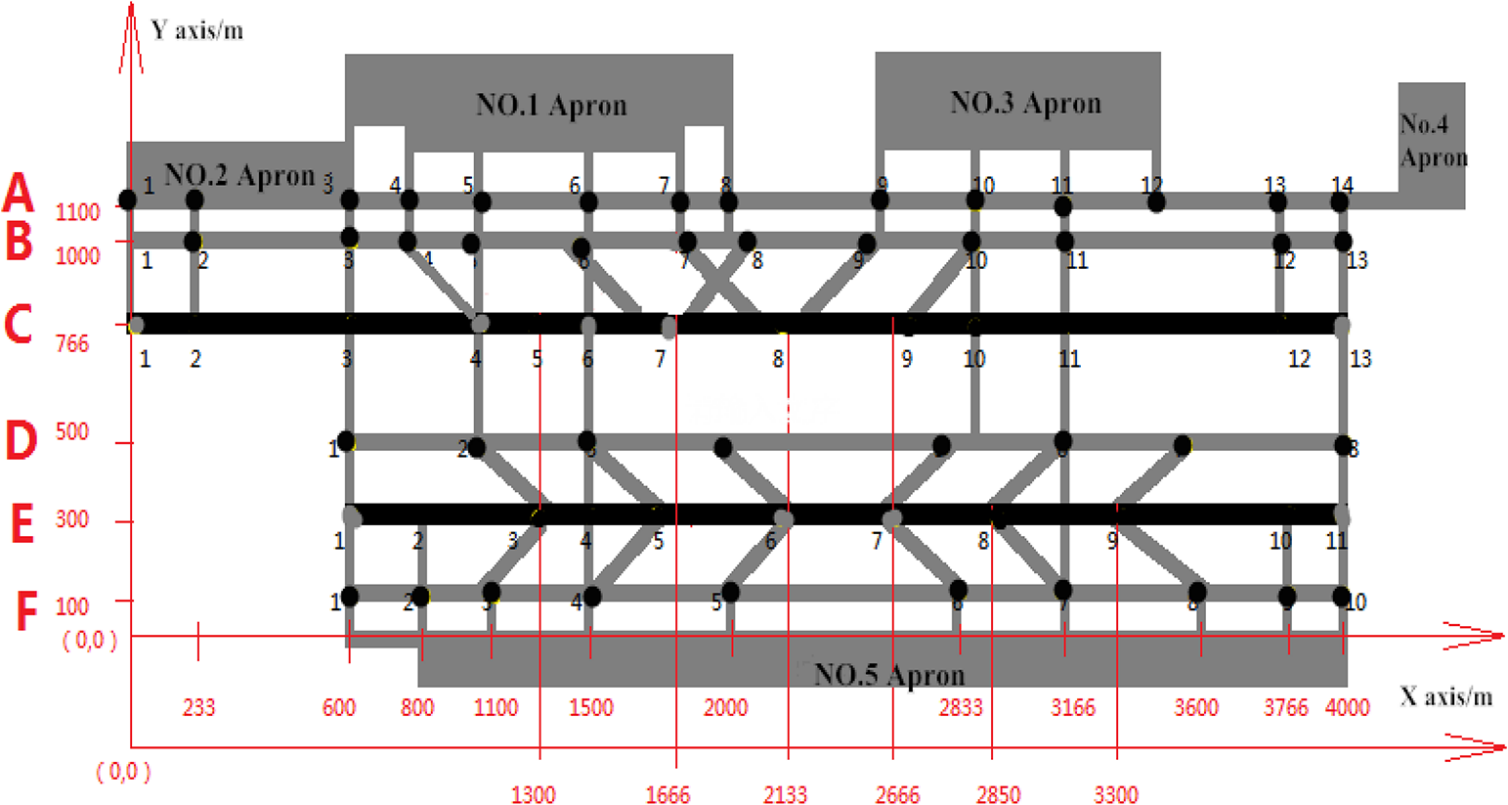
The sliding network of Chongqing Jiangbei Airport.

The sliding network is shown in Fig 5. Each runway is unidirectional. The taxiways and runways are named *A*, *B*, *C*, *D*, *E*, *F* from north to the south, totally six, and the name of the node in the abeam links is consist of number and name from west to east. In this network Fig, the nodes intersected with the aprons and runways are the collection of starting nodes for the flight, and the terminal nodes of all flights are *C*1 or *E*1. Because the runway occupancy cost is relatively large and likely to cause potential risks, so make a rule that it should not expend two consecutive nodes on the runway, so as to avoid the impact on airport operations.

#### Initial path solution

The initial flight data is listed in Table 4, coming from Chongqing Jiangbei Airport (shown in S1 Dataset). It is the simulant data of a certain period. Make an experiment of these arrival or departure flights on C++ platform. The starting time, starting node, terminal node and the speed are listed in Table 4 (The nodes in the runway should not be the consecutive expended nodes).

**Table 4:** Initial flight data.

	Flight Number	Starting Time	Starting Point	Terminal Point	Type	Average speed m/s	Arrival or Departure(A or D)
1	CA3133	7:53:00	C4	F6	A320	5	A
2	CA8894	8:00:00	E8	F9	A320	5	A
3	3U3183	8:04:00	A9	C1	A319	5	D
4	CZ6235	8:08:00	C7	A14	B747	7	A
5	CA5236	8:04:00	F6	E1	A330	6	D
6	3U2158	8:03:40	A10	C1	B747	7	D
7	PN6273	8:10:00	A11	E1	B737	5	D
8	3U1478	8:05:00	C4	F6	A320	5	A
9	CZ6547	8:30:00	E9	A3	A320	5	A
10	CA3813	8:15:00	A10	C1	A319	5	D

The initial paths of the flights were calculated and shown in Table 5 (When there is a conflict at a certain node for the aircraft, its subsequent path is no longer given). Here, the A320, A319, B737 are light flights, their average speed is written as 5m/s, A330 is medium flight and B747 is heavy flight, their average speeds are written as 6m/s and 7m/s respectively. The length of each path is calculated based on the coordinates of the nodes. The specific coordinates refer to Fig 5, the time of aircraft sliding and its starting and ending point refer to the Table 4.

**Table 5:** The sliding plan of each aircraft.

Flight Number	Initial Path
CA3133	(C4,7:53:00) → (D2,7:53:53) → (D4,7:56:53) → (E6,7:56:41) → (E7,7:59:28) →(F6,8:00:20) →(**F7,8:01:27**)
CA8894	(E8,8:00:00) → (**F7,8:01:14)**
3U3183	**(A9,8:04:00)**
CZ6235	(C7,8:08:00) → (B8,8:08:58) → (**B10,8:10:58)**
CA5236	(F6,8:04:00) → (F1,8:10:12) → (E1,8:10:45)
3U2158	(A10,8:03:40) → (**A9,8:04:05**) → (B9,8:04:19) → (B1,8:10:40) → (C1,8:11:13)
PN6273	(A11,8:10:00) → (B11,8:10:20) → (**B10,8:11:25)**
3U1478	(C4,8:05:00) → (D2,8:05:53) → (D3,8:07:13) → (D4,8:08:53) → (E6,8:09:41) → (E7,8:11:28) →(F6,8:12:20)
CZ6547	(E9,8:30:00) → (D7,8:31:12) →(D2,8:32:05) →(C4,8:32:58) →(B4.8:34:14) → (B3,8:34:54) → (A3,8:35:14)
CA3813	(A10,8:15:00) → (A9,8:15:34) → (B9,8:15:54) → (B1,8:24:47) → (C1,8:25:34)

#### Conflict adjustment

As can be seen from the Table 5, there is conflict in the initial paths of some certain flights (shown in bold). Then by means of the improved A * algorithm, the conflict avoidance module is applied to make further optimization of sliding path to reduce losses from conflict. In the waiting process, the resources occupied by the flight and the fuel consumption are less, so the weight coefficient of waiting cost could be set a litter smaller. Therefore, the weight coefficient of waiting cost is *y* = 0.2, the weight coefficient of sliding cost is *x* = 0.8.

#### Tailgating conflict adjustment

3U2158:(A10,8:03:40)→(A9,8:04:05)→(B9,8:04:19)→(B1,8:10:40)→(C1,8:11:13)

3U3183:(A9,8:04:00)→(B9,8:04:20)→(B5,8:09:13)→(B1,8:12:53)→(C1,8:12:57)

The sliding path is the same of these two flights after passing the node A9, but the time interval is 5s, it does not meet the 20s safety interval requirement in Table 1. The light flight 3U3183 arrives at the node A9 firstly, while the heavy flight 3U2158 arriving 5s later and its speed is faster than light flight, so there is tailgating conflict. The potential conflict of node A9 is shown in Table 6.

**Table 6:** The potential conflict of node A9.

Node Number	Flight Number	Type	Arrival Time	Father Node	Successor Node
A9	3U2158	H	8:04:05	A10	B9
A9	3U3183	L	8:04:00	No	B9

Heavy flight has higher priority, so flight 3U2158 goes on sliding according to original plan. Detect that there are two successive nodes A9 and B9, and the path is coincident, so flight 3U3183should wait before the node, and the waiting time is time interval (5s) + safety time interval (20s), totally 25s. The length of path is 3000m. So,

D(1)=3000*0.8/5+25*0.2=485 (8)

The suboptimal node is A10, the length of sliding path is more than initial path 468m, and there is no need conflict waiting. So the value of suboptimal node is:

D(2)=(3000+468)*0.8/5+0*0.2=554.88 (9)

*D*(1) < *D*(2), the min*D*(*m*) is *D*(1). It is needed to choose the optimal node path, hold the position 25s until conflict over. New path of flight 3U3183 is:

3U3183:(A9,8:04:25)→(B9,8:04:55)→(B5,8:09:48)→(B1,8:13:28)→(C1,8:13:32)

The conflict adjustment of node A9 is shown in Table 7.

**Table 7:** Conflict adjustment of node A9.

Node Number	Flight Number	Type	Arrival Time	Father Node	Successor Node
A9	3U2158	H	8:04:05	A10	B9
A9	3U3183	L	8:04:30	No	B9

After adjustment, the heavy flight goes firstly and the light flight waiting at the position, so the tailgating conflict could be avoided.

#### Head conflict adjustment

CZ6235:(C7,8:08:00)→(B8,8:08:58)→(B9,8:10:14)→(B10,8:10:58)→(A10,8:11:12)→(A14,8:13:57)

PN6273:(A11,8:10:00)→(B11,8:10:20)→(B10,8:11:25)→(B9,8:12:00)→(B8,8:14:13)→(C7,8:15:34)→(C6,8:16:04)→(D3,8:16:57)→(D1,8:19:57)→(E1,8:20:37)

The arriving time interval is 27s at node B10, if these two flights keep on sliding, it may lead to head conflict. Because of the heavy flight with higher priority, so it needs to make adjustment of light flight PN6273. The potential conflict of node B10 is shown in Table 8.

**Table 8:** The potential conflict of node B10.

Node Number	Flight Number	Type	Arrival Time	Father Node	Successor Node
B10	CZ6235	H	8:10:58	B9	A10
B10	PN6273	L	8:11:25	B11	B9

The waiting time at the original node is 3s, and the length of path is 2781m. So,

D(1)=2781*0.8/5+3*0.2=445.56 (10)

The suboptimal node is A10, and updates the new path data. The new path after choosing suboptimal node of the flight PN6273 is:

PN6273:(A11,8:10:00)→(A10,8:11:05)→(A9,8:11:39)→(B9,8:12:00)→(B8,8:14:13)→(C7,8:15:34)→(C6,8:16:04)→(D3,8:16:57)→(D1,8:19:57)→(E1,8:20:37)

There is no conflict in the suboptimal node path, the length is 2781m.

D(2)=2781*0.8/5+0*0.2=444.96 (11)

*D*(2) < *D*(1), so the min*D*(*m*) is *D*(2). Choose the suboptimal node path. After adjustment, the path of flight PN6273 will not pass the node B10 anymore, and head conflict is avoided.

#### Intersection point conflict

CA3133:(C4,7:53:00)→(D2,7:53:53)→(D4,7:56:53)→(E6,7:56:41)→(E7,7:59:28)→(F6,8:00:20)→(F7,8:01:27)→(F8,8:02:53)

CA8894:(E8,8:00:00)→(F7,8:01:14)→(F9,8:03:14)

These two flights both need to pass the node F7, the time interval is 13s, does not meet the safety interval. So there is an intersection point conflict. The potential conflict of node F7 is shown in Table 9. Because they are both light flights, the first arriving flight with higher priority, so the flight CA8894 keeps going on its original path, make adjustment of flight CA3133.The system detects that there does not exist suboptimal node, so it should stay in the position. After adjustment, the new path is:

CA3133:(C4,7:53:00)→(D2,7:53:53)→(D4,7:56:53)→(E6,7:56:41)→(E7,7:59:28)→(F6,8:00:37)→(F7,8:01:34)→(F8,8:03:10)

**Table 9:** The potential conflict of node F7.

Node Number	Flight Number	Type	Arrival Time	Father Node	Successor Node
F7	CA3133	L	8:01:27	F6	F8
F7	CA8894	L	8:01:14	E8	F8

The conflict adjustment of node F7 is shown in Table 10. After adjustment, the flight CA3133 passes the node F7, and the time interval of former flight is 20s, the conflict is avoided.

**Table 10:** Conflict adjustment of node F7.

Node Number	Flight Number	Type	Arrival Time	Father Node	Successor Node
F7	CA3133	L	8:01:34	F6	F8
F7	CA8894	L	8:01:14	E8	F8

By conflict detection module, a total of three flights were adjusted, they were CA3133, 3U3183 as well as PN6273. The final paths after conflict detection were shown in Table 11. And the paths being adjusted were shown in bold.

**Table 11:** The final optimal sliding paths of improved A* algorithm.

Flight Number	Adjusted Path
***CA3133***	***(C4*, *7*:*53*:*00) → (D2*, *7*:*53*:*53) → (D4*, *7*:*56*:*53) → (E6*, *7*:*56*:*41) → (E7*, *7*:*59*:*28) →(F6*, *8*:*00*:*37) →(F7*, *8*:*01*:*34) →(F8*, *8*:*03*:*10)***
CA8894	(E8, 8:00:00) → (F7, 8:01:14) → (F9, 8:03:14)
***3U3183***	***(A9*, *8*:*04*:*30) → (B9*, *8*:*04*:*55) → (B5*, *8*:*09*:*48) → (B1*, *8*:*13*:*28) → (C1*, *8*:*13*:*32)***
CZ6235	(C7, 8:08:00) → (B8, 8:08:58) → (B10, 8:10:58) → (A10, 8:11:12) → (A14, 8:13:57)
CA5236	(F6, 8:04:00) → (F1, 8:10:12) → (E1, 8:10:45)
3U2158	(A10, 8:03:40) → (A9, 8:04:05) → (B9, 8:04:19) → (B1, 8:10:40) → (C1, 8:11:13)
***PN6273***	***(A11*, *8*:*10*:*00) → (A10*, *8*:*11*:*05) → (A9*, *8*:*11*:*39) → (B9*, *8*:*12*:*00) → (B8*, *8*:*14*:*13) → (C7*, *8*:*15*:*34) → (C6*, *8*:*16*:*04) →(D3*, *8*:*16*:*57) → (D1*, *8*:*19*:*57) → (E1*, *8*:*20*:*37)***
3U1478	(C4, 8:05:00) → (D2, 8:05:53) → (D3, 8:07:13) → (D4, 8:08:53) → (E6, 8:09:41) → (E7, 8:11:28) → (F6, 8:12:20)
CZ6547	(E9, 8:30:00) → (D7, 8:31:12) → (D2, 8:32:05) → (C4, 8:32:58) → (B4, 8:34:14) → (B3, 8:34:54) → (A3, 8:35:14)
CA3813	(A10, 8:15:00) → (A9, 8:15:34) → (B9, 8:15:54) → (B1, 8:24:47) → (C1, 8:25:34)

### Analysis

After conflict adjustment, these ten flights meet the safety interval and avoid conflict. So, they are the final optimal sliding paths. Besides, the sliding paths could be modified in real time according to the circumstances. Since each node records the flight time data, it also shows the position of each flight on the network. So as to viewing and comparing timely and avoid risk.

Here, the initial path got by the A* algorithm is also given in Table 12.

**Table 12:** The sliding plan of A* algorithm.

Flight Number	Initial Path
CA3133	(C4, 7:53:00) → (D2, 7:53:53) → (D4, 7:56:53) → (E6, 7:56:41) → (E7, 7:59:28) →(F6, 8:00:20) →(F7, 8:01:27) →(F8, 8:02:53)
CA8894	(E8, 8:00:00) → (F7, 8:01:14) → (F9, 8:03:14)
3U3183	(A9, 8:04:00) → (B9, 8:04:20) → (B5, 8:09:13) → (B1, 8:12:53) → (C1,8:12:57)
CZ6235	(C7, 8:08:00) → (B8, 8:08:58) → (B10, 8:10:58) → (A10, 8:11:12) → (A14, 8:13:57)
CA5236	(F6, 8:04:00) → (F1, 8:10:12) → (E1, 8:10:45)
3U2158	(A10, 8:03:40) → (A9, 8:04:05) → (B9, 8:04:19) → (B1, 8:10:40) → (C1, 8:11:13)
PN6273	(A11, 8:10:00) → (B11, 8:10:20) → (B10, 8:11:25) → (B9, 8:12:00) → (B8, 8:14:13) → (C7, 8:15:34) → (C6, 8:16:04) → (D3, 8:16:57) → (D1, 8:19:57) → (E1, 8:20:37)
3U1478	(C4, 8:05:00) → (D2, 8:05:53) → (D3, 8:07:13) → (D4, 8:08:53) → (E6, 8:09:41) → (E7, 8:11:28) →(F6, 8:12:20)
CZ6547	(E9, 8:30:00) → (D7, 8:31:12) → (D2, 8:32:05) → (C4, 8:32:58) → (B4, 8:34:14) → (B3, 8:34:54) → (A3, 8:35:14)
CA3813	(A10, 8:15:00) → (A9, 8:15:34) → (B9, 8:15:54) → (B1, 8:24:47) → (C1, 8:25:34)

On one hand, according to Table 11 and Table 12, make a comparison of A* algorithm and the improved A* algorithm, we can conclude that:

1. For flight CA3313: in A*algorithm, the time of the flight passing the terminal node F8 is 8:02:53, after adjustment, the time passing the node F8 is 8:03:10, later than A* algorithm by 17s;
2. For flight 3U3183: in A* algorithm, the time of the flight passing the terminal node C1 is 8:12:57, passing the node A9 is 8:04:00, after adjustment, the time passing the node C1 is 8:13:32, passing the node A9 is 8:04:35. Total sliding time is the same 537s. But, the revised path avoided tailgating conflict;
3. For flight PN6273: Compare the path got by the improved algorithm with the path got by A* algorithm, though the sliding time is the same, the revised flight path avoided the node B10, re-selected a new route to avoid the conflict.
4. In summary, as can be seen from above comparison, although in some cases, the optimization paths made the flight sliding time longer, it ensured the safety of the flight very well, and avoided the serious accidents, reduced the loss of flight from long-term perspective; On the contrary, there were also some optimization paths which the time of sliding is not longer and even shorter. So the improved A* algorithm in this paper was feasible, which made paths optimized, conflict avoided and ensured the safety.

**Fig 6:**
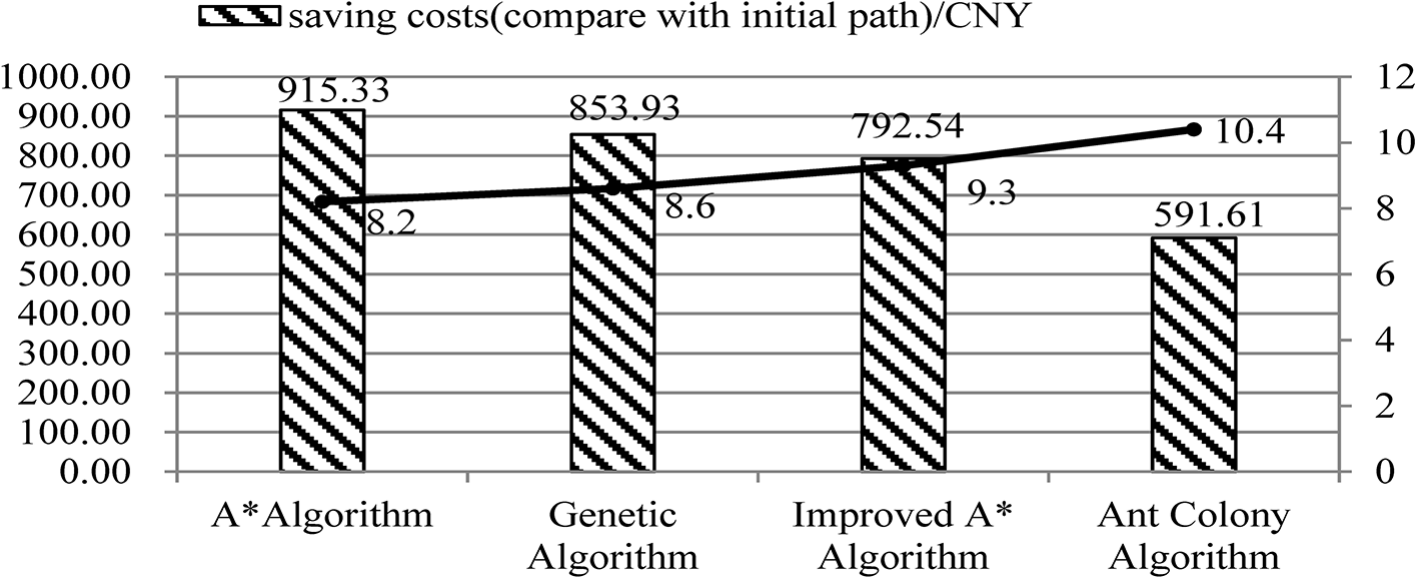
The comparison of algorithms.

In order to verify the superiority of the improved A* algorithm, make comparison with other algorithms. Select the genetic algorithm in literature 10 to compare with general A* algorithm and prove the high efficiency of A* algorithm; Meantime, compare the improved A* algorithm with ant colony algorithm in literature 18, both of them considered the conflict detection problem. Make comparison of these four algorithms. Run these four algorithms in C++ platform using the initial flight data in this paper, get the sliding paths and sliding time. The results are as follows:

1. Compare with initial sliding path, the sliding time of A* algorithm is 4564s, reduce by 164s; the sliding time of genetic algorithm is 4575s, reduce by 153s. The operation time of A* algorithm is 8.4s, the genetic algorithm is 9.1s. We can conclude that the A* algorithm has higher efficiency. So, on the basis of A* algorithm, add conflict detection strategy into it to make improvement and ensure safety.
2. Compare improved A* algorithm with ant colony algorithm who also considered conflict detection. Sliding time of improved A* algorithm is 4586s, the ant colony algorithm is 4622s, algorithm operation time is 12.3s and 14.4s respectively.
3. Treat the Boeing 737 as an example, the sliding fuel consumption of the aircraft is 2.85t per hour. Suppose the price of aviation oil is 7050CNY/t, after optimization, the cost of aviation oil could be saved a lot. The comparison of these algorithms is shown in Fig 6. (Here, 915.33 = 7050*2.86/3600*164, the rest can be done in the same manner.)

As can be seen from the Fig 6, A*algorithm and genetic algorithm are optimized shortest path algorithm, but the A* algorithm has high efficiency with shortest sliding time and algorithm operation time. The saving costs increased by 6.4% ((915.33–859.93)/859.93 = 6.4%), algorithm operation time increased by 4.9% ((8.6–8.2)/8.2 = 4.9%). Though the ant colony also takes into consideration of conflict detection, but its results is not well as improved A * algorithm. The saving costs of improved A* algorithm increased by 33.9% ((792.54–591.61)/591.61 = 33.9%), the algorithm operation time increased by 10.6% ((10.4–9.3)/10.4 = 10.6%) compared with ant colony algorithm. All in all, the improved A* algorithm proposed in this paper has feasibility and superiority, and ensure the safety at the same time.

## Conclusions

In this paper, the taxiway path planning and conflict avoid problems are studied to look for the optimal paths which could avoid conflict intelligently, balance the delay cost and sliding path length. The concept of node schedule is proposed innovatively, and the flight data for each node combined with flight speed is built. Regard it as the basis for conflict detection. Add conflict detection strategy into the algorithm and apply multi-objective optimization function to take conflict avoidance measures, make the choice of waiting or choosing another new path according to it, so the improved A* algorithm is created. Finally, the optimal sliding paths are generated which has shortest sliding length as well as ensures the safety. By making instance analysis, verify the feasibility and effectiveness of the model:

1. By making comparison of the general A* algorithm with genetic algorithm, the saving costs increased by 6.4%, and the algorithm operation time saved by 4.9%. So the A* algorithm has higher efficiency.
2. Compare the improved A* algorithm with the general A* algorithm, the sliding time is longer than A* algorithm 22s, algorithm operation time longer 1.1s. But it ensures the safety of flight and passengers, and avoids the happening of accident.
3. Compared the ant colony algorithm with the improved A* algorithm, though it also takes into consideration of conflict detection, but its results is not well as improved A * algorithm. The saving costs of improved A* algorithm increased by 33.9%, the algorithm operation time increased by 10.6%.

So the model and algorithm established in this paper are feasible and effective, which could provide a reference for aircraft to pre-arrange paths.

## Supporting Information

S1 Dataset Flight data. (XLSX)
